# A novel mutation in ryanodine receptor 2 (*RYR2*) genes at c.12670G>T associated with focal epilepsy in a 3-year-old child

**DOI:** 10.3389/fped.2022.1022268

**Published:** 2022-10-19

**Authors:** Junji Hu, Xueping Gao, Longchang Chen, Tianshu Zhou, Zhaoli Du, Jinghan Jiang, Lei Wei, Zhijun Zhang

**Affiliations:** ^1^ Department of Neurology, Zibo Changguo Hospital, Zibo, China; ^2^ Yinfeng Gene Technology Co., Ltd., Jinan, China; ^3^ The First Clinical College, Hubei University of Medicine, Shiyan, China; ^4^ Department of Center for Reproductive Medicine, TaiHe Hospital, Hubei University of Medicine, Shiyan, China

**Keywords:** ryanodine receptor 2, catecholaminergic polymorphic ventricular tachycardia, epilepsy, genetic focal epilepsy, whole exome sequencing

## Abstract

**Background:**

Ryanodine receptor 2 (*RYR2*) encodes a component of a calcium channel. *RYR2* variants were well-reported to be associated with catecholaminergic polymorphic ventricular tachycardia (CPVT), but rarely reported in epilepsy cases. Here, we present a novel heterozygous mutation of *RYR2* in a child with focal epilepsy.

**Methods:**

At the age of 2 years and 7 months, the patient experienced seizures, such as eye closure, tooth clenching, clonic jerking and hemifacial spasm, as well as abnormal electroencephalogram (EEG). Then, he was analyzed by whole-exome sequencing (WES). The mutations of both the proband and his parents were further confirmed by Sanger sequencing. The pathogenicity of the variant was further assessed by population-based variant frequency screening, evolutionary conservation comparison, and American Association for Medical Genetics and Genomics (ACMG) scoring.

**Results:**

WES sequencing revealed a novel heterozygous truncating mutation [c.12670G > T, *p*.(Glu4224*), NM_001035.3] in *RYR2* gene of the proband. Sanger sequencing confirmed that this mutation was inherited from his mother. This novel variant was predicted to be damaging by different bioinformatics methods. Cardiac investigation showed that the proband had no structural abnormalities, but sinus tachycardia.

**Conclusion:**

We proposed that RYR2 is a potential candidate gene for focal epilepsy, and epilepsy patients carried with RYR2 variants should be given more attention, even if they do not show cardiac abnormalities

## Introduction

Ryanodine receptor 2 (*RYR2*) gene, localized on chromosome 1q43, encodes a component of a calcium channel. RYR2 is localized to the sarcoplasmic reticulum and regulates intracellular calcium ion release and cardiac contraction (1). The RYR2 protein is ubiquitously distributed and highly expressed in myocardium, placenta and brain (cerebellum and hippocampus) tissues (2). It has been well studied that *RYR2* gene is discovered as a candidate pathogenic gene in catecholaminergic polymorphic ventricular tachycardia (CPVT, OMIM: 604772) (3, 4). CPVT is a rare hereditary arrhythmogenic disorder, which is triggered by exercise or emotional stress (5). Phenotypic manifestation in CPVT1 is extremely heterogeneous. The main clinical features of CPVT are various forms of ventricular arrhythmias that are absent under resting conditions but present only during physical exercise, excitement, or catecholamine administration. It is reported that CPVT is one of the most common causes of syncope and sudden death in adolescents and children (6). Approximately 50% of CPVT probands carried with *RYR2* mutations in a Dutch cohort presented with seizures during the course of the syncopal events (7). Recently, it was reported that a *RYR2* mutation at c.229G > A was identified in a family with CPVT, but a carrier presented with generalised seizures without any cardiac abnormalities (8). However, the relationship between *RYR2* gene and epilepsy is not well determined.

Additionally, This study identified a novel mutation of *RYR2* in a 3-year and 4-month-old boy with focal epilepsy using the whole-exome sequencing (WES), supplementing the phenotypic spectrum of *RYR2* mutations. The mutation was supposed to impact the structure and function of the RYR2 protein. We, therefore, aimed to clarify the pathogenicity of this variant.

## Materials and methods

### Subjects

**Figure 1:**
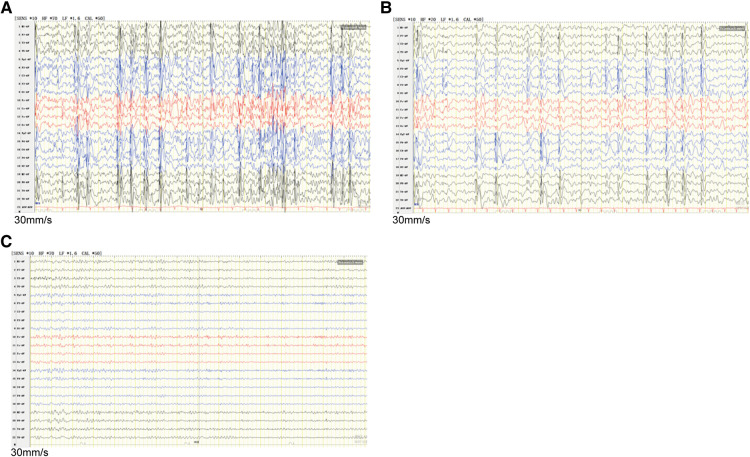
EEG discharges in the proband and his mother. (**A**) Interictal EEG recorded of the proband during waking showed generalized spike-slow wave complexes. (**B**) The interictal EEG of the proband during sleep showed runs of spike-slow wave complexes arising from bilateral temporal regions. (**C**) The mother's EEG showed epileptiform discharges in the midline area. EEG, Electroencephalography.

The study protocol was approved by the Ethics Committee of Zibo Changguo Hospital. We have obtained consent from the parents to publish this case. A 3-year and 4-month-old boy presenting with a series of seizures during sleep for the first time at the age of 2 years and 7 months was referred to hospital in June, 2020. The exhibiting symptoms of the patient included expiration, closed eyes, and forced shaking of the left upper limb, which resolved in 1–2 min. These symptoms were mainly concentrated on the left face and left upper limb. He had another seizure during sleep after a month. After taking levetiracetam tablets (25 mg/kg) every day, the above symptoms did not appear again. However, at the age of 3 years and 4 months, he relapsed with unknown reasons during mealtime. In addition to the above symptoms, he also had left-sided facial muscle spasm and clenching of teeth, lasting for dozens of seconds. According to the description of the patient's parents, the patient's language fluency was relatively poor since childhood, but there was no obvious abnormality in his comprehension and logical thinking ability. He was not fluent in speech and only speaks in simple sentences at the first visit. The patient never described any cardiac symptoms, such as chest pain, palpitations, dizziness, or shortness of breath. Video electroencephalogram (VEEG) result showed abnormal electroencephalogram (EEG): generalized spike-slow wave complexes during waking, spike-slow wave complexes in the bilateral centrotemporal, parietotemporal, mid-temporal, posterior temporal regions during sleep (Figures 1A,B). No obvious abnormality was found on brain CT scan. The family rejected the magnetic resonance imaging (MRI) scan. The electrocardiogram (ECG) showed sinus tachycardia (Supplementary Figure S1A), and the color Doppler ultrasound showed no abnormality in the proband's heart. The patient's mother showed an abnormal EEG with epileptiform discharges in the midline region (Figure 1C) and likewise an ECG showing sinus tachycardia (Supplementary Figure S1B), and abnormal left ventricular filling was shown by the color Doppler ultrasound.

### Whole-exome sequencing (WES) analysis

WES sequencing was tested by Yin Feng Gene Technology Co., Ltd. (Jinan, China). Briefly, genomic DNA was extracted from peripheral blood for each sample using Magnetic Universal Genomic DNA Kit (TIANGEN, China). The quality and quantity of each DNA sample were detected by 1% agarose gel electrophoresis, NanoPhotometer (IMPLEN, CA, USA) and Qubit® 3.0 Flurometer (Life Technologies, CA, USA). Then, DNA libraries were prepared using Illumina standard protocol. According to the manufacturer's instructions, the exome was captured using IDT xGen Exome Research Panel v1.0 (Integrated DNA Technologies, Coralville, Iowa, USA), and sequenced using Illumina Novaseq 6000 platform (Illumina Inc., San Diego, CA, USA) with a depth of 100-fold. The data obtained by WES sequencing was then used for mutation hazard prediction, genotype-phenotype correlation analysis and mutation screening.

### Sanger sequencing

To further verify the existence of the mutation of *RYR2* [c.12670G > T/*p*.(Glu4224*)] in proband and their parents, DNA was extracted using TIANGEN Universal DNA Purification kit (TIANGEN) and PCR amplified using the following primers of RYR2 (NM_001035.2): 5′-CCGACTTCAGACTTTTCTGGAATTA-3′ and 5′-AAGTGCAAGAGGGTCATGAAAATAC-3′. The product was sequenced by Yin Feng Gene Technology Co., Ltd. using ABI 3730xl DNA Analyzer (ABI, USA).

### Bioinformatics analysis

After sequencing, bcl2fastq software (Illumina) was performed for base-call file conversion and emultiplexing. The resulting fastq data was analyzed by internal quality control software to remove low quality reads, and then burrows Wheeler comparator (BWA) software (9) was performed to align to human reference genome (hg37), Sambamba tools were used to mark the duplicated reads (10). Single nucleotide variants (SNVs) and indels identification were carried out using GATK software (11), and ANNOVAR software (12) was used to annotate the detected mutation sites.

SNVs/Indels were compared with the 1000 Genomes Project, ExAC, and gnomAD databases. MutationTaster (MT), SIFT, PolyPhen-2, GERP++, CADD, Revel score, and M-CAP were performed to predict the hazards of mutations. According to the guidelines of American College of Medical Genetics and Genomics (ACMG), the pathogenicity of variations was classified. MEGA-X software was used for multi-species protein sequence alignments, and SWISS-MODEL was performed to construct the 3D protein structure of wild-type or mutated RYR2 protein (13).

STRING (https://string-db.org/) was used to build protein-protein interaction (PPI) network associated with *RYR2*. The Gene Ontology (GO) classification and Kyoto Encyclopedia of Genes and Genomes (KEGG) pathway enrichment analysis of the target genes in PPI network were downloaded in STRING and was plotted by https://www.bioinformatics.com.cn, a free online platform for data analysis and visualization.

## Results

### WES results analysis

WES sequencing was performed to screen for mutants in the patient. Among the mutations with a frequency <0.001, we found a heterozygous truncating mutation [c.12670G > T/*p*.(Glu4224*), NM_001035.3] of *RYR2* that was not present in the ExAC, 1000 Genomes, or gnomAD database. The truncating mutation *p*.(Glu4224*) was suggested to be damaging by the web-based prediction tools (CADD, MutationTaster, DANN, and FATHMM). VarCards (http://varcards.biols.ac.cn/) was used to analyze the hazards of this variation of *RYR2*. The pathogenicity identification score of VarCards ranges from 0 to 1, with higher scores indicating stronger pathogenicity (14). The pathogenicity identification score of *RYR2* mutation c.12670G > T was 1, indicating a stronger pathogenicity. According to the classification of sequence variants recommended by the ACMG, this novel truncated mutation in the *RYR2* gene fulfilled the criteria for uncertain significance (VUS) (PVS1-strong + PM2_Supporting), which could be sub-classified as “VUS favor P” (15). In addition, no other significant variants, including copy number variants (CNVs), were found in other epilepsy related genes.

### Mutation detection

**Figure 2:**
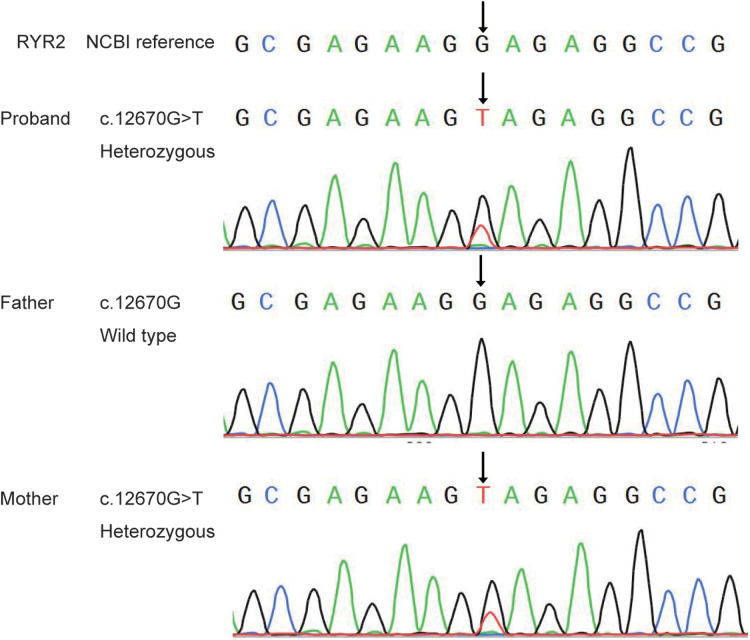
Sanger sequence analysis of the novel mutation of RYR2 gene in the proband and his parents.

Sanger sequencing showed that the mutation of *RYR2* gene c.12670G > T/*p*.(Glu4224*) was inherited from the patient's mother, his father's gene was wild-type (Figure 2). However, there was no history of epilepsy in this family. But the ECG revealed sinus tachycardia in both the patient and his mother.

### Pathogenic assessment

**Figure 3:**
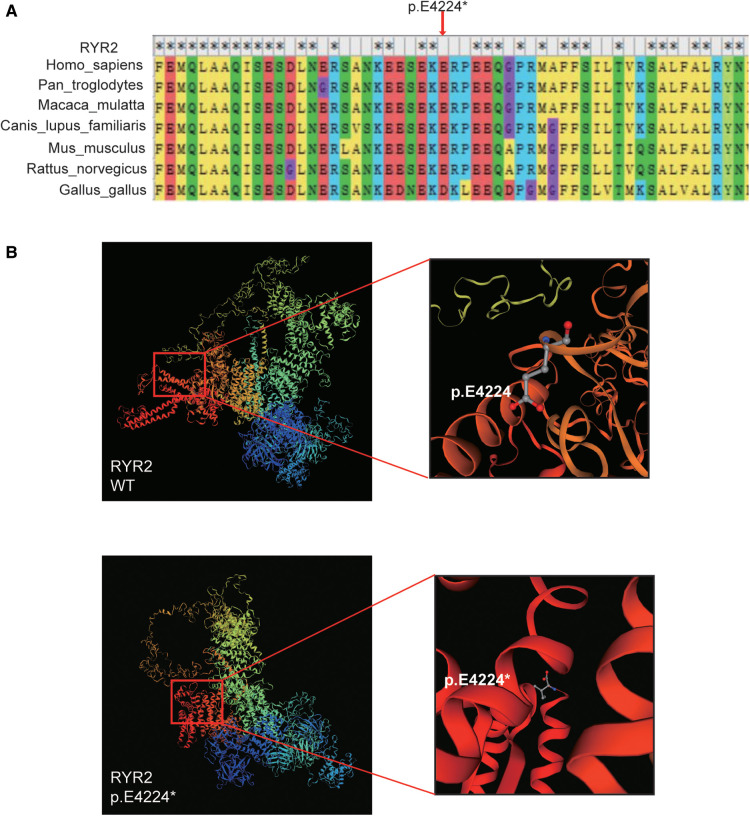
Bioinformatics analysis. (**A**) The multi-species alignment near the site of *p*.E4224. (**B**) The variant type of *p*.E4224* and wild-type tertiary structures of RYR2 protein predicted by SWISS-MODEL.

The variant of *RYR2* c.12670G > T/*p*.(Glu4224*) was not present in the SNP database, the 1000 Human Genome Database, gnomeAD, ClinVar, or the HGMD database. As mentioned above, the hazard of this truncating mutation *p*.(Glu4224*) was predicted by the web-based prediction tools. The truncating mutation *p*.(Glu4224*) may cause malformations of RYR2 protein, leading to insufficient functional haploids. We performed a multispecies alignment of the amino acids encoded by *RYR2*, including *Homo sapiens* (human), *Pan troglodytes* (Chimpanzee), *Macaca mulatta* (Rhesus macaque), *Canis lupus familiaris* (Dog), *Mus musculus* (Mouse), *Rattus_norvegicus* (Rat), and *Gallus gallus* (Chicken). As shown in Figure 3A, the results of alignment near the *p*.(Glu4224*) variant site revealed the conservation of the sequence. Next, the 3D protein structure of the truncating variant was further predicted by SWISS-MODEL (Figure 3B). The predicted models showed that once amino acid 4224 changes from a glutamate to a stop codon, the RYR2 protein was forced to be truncated and its 3D protein structure was subsequently changed (Figure 3B). These changes may lead to altered protein function, resulting in damage. Collectively, this novel truncating mutation [*p*.(Glu4224*)] of *RYR2* was identified as the likely pathogenic variant for the patient.

**Figure 4:**
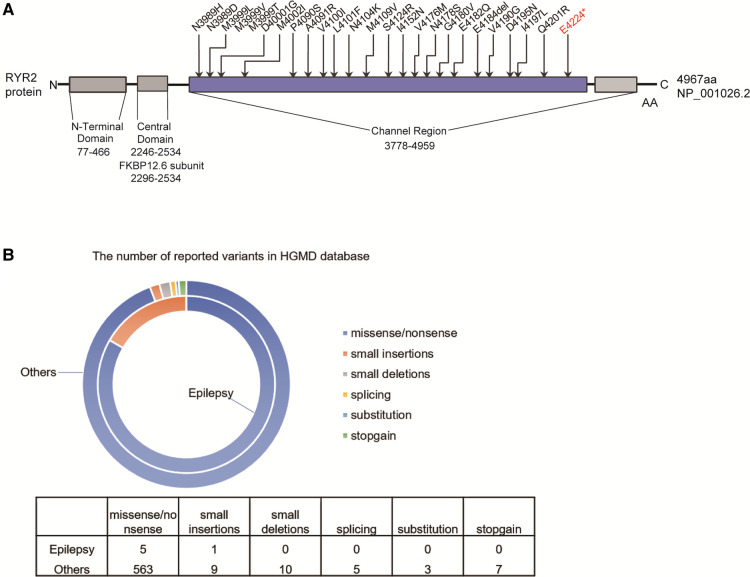
The reported RYRA2 variants. (**A**) Schematic diagram of the pathogenic or likely pathogenic mutations presented in the ClinVar database in the exon 90 of the RYR2 gene. (**B**) The number of reported RYR2 variants associated with epilepsy (small deletions, small insertions, splicing, stopgain, and missense/nonsense variants) in the HGMD.

The variant [c.12670G > T/*p*.(Glu4224*)] of *RYR2* was located in the exon 90 of *RYR2* gene, which is also the channel region of RYR2 protein. We counted the pathogenic or likely pathogenic variants located in exon 90 of the *RYR2* gene presented in the ClinVar database (Figure 4A). Importantly, 12 of these variants have been reported to be correlated with CPVT.

Total of 597 variants in *RYR2* gene are included in the HGMD database, of which 6 variants have been reported to be associated with seizures (Figure 4B). Among 6 different variants of the *RYR2* gene, there 5 missense/nonsense variants and 1 small insertion. Variants in the *RYR2* gene are widely distributed in almost every exon (Supplementary Table S1).

### Protein-protein interaction network (PPI) and functional annotation analysis

**Figure 5:**
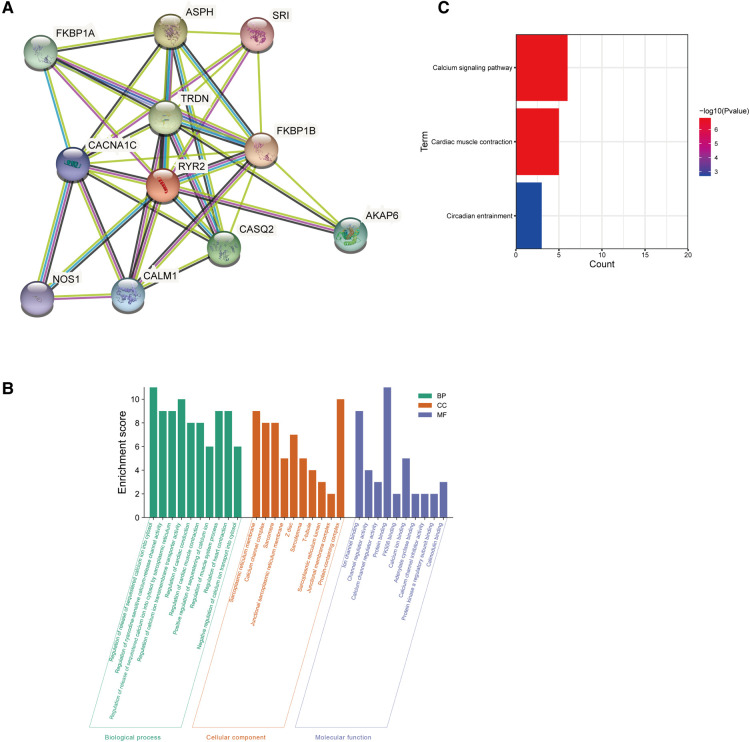
Go and KEGG enrichment analysis of the PPI network. (**A**) PPI network analysis of interacting genes with RYR2. Circles are used to represent nodes, and lines are used to represent edges. (**B**) GO terms of target genes. (**C**) KEGG pathways.

The PPI network analysis of interacting genes with *RYR2* was produced by STRING. The PPI network consisted of 11 nodes and 36 edges (Figure 5A). The top two proteins with higher degrees are FKBP1B (degree = 9), CACNA1C (degree = 9) and TRDN (degree = 9). Then, GO enrichment analysis suggesting that these genes significantly enriched in regulation of release of sequestered calcium ion into cytosol and regulation of calcium ion transmembrane transporter activity of BP, protein-containing complex and sarcoplasmic reticulum membrane of CC, protein binding and ion channel binding of MF (Figure 5B). KEGG pathway enrichment analysis showed that these target genes played an essential role in Calcium signaling pathway and Cardiac muscle contraction (Figure 5C).

### Follow-up

The child had relapse 8 months after the first medication, which may be caused by inadequate levetiracetam due to physical development. The patient got seizure-free with combination of levetiracetam (25 mg/kg/day) and oxcarbazepine (23 mg/kg/day) after this diagnosis. One and a half years after diagnosis, the follow-up found that the patient's condition was well controlled, and the language fluency was better than before.

## Discussion

In the present study, we used WES to identify a novel truncating mutation [c.12670G > T/*p*.(Glu4224*)] of *RYR2* in a child with focal epilepsy. Since there is currently no evidence for haploinsufficiency of the RYR2 gene in the ClinGen database, the weight of criterion PVS1 was decreased one level (PVS1-strong). Due to the recent proposal by the ClinGen Sequence Variant Interpretation (SVI) Working Group to decrease the weight of criterion PM2, based on currently available evidence, this variation has been downgraded from likely pathogenicity to uncertain significance (PVS1-strong + PM2_Supporting). However, as a nonsense mutation, multiple bioinformatics methods predict this variant to be detrimental, including CADD, MutationTaster, DANN, FATHMM, and VarCards.

*RYR2* is located on chromosome 1q43 and encodes a ryanodine receptor that is one of the components of a calcium channel, controlling intracellular calcium release and cardiac contraction. The RYR2 protein consists of a tetramer of ryanodine receptor proteins and a tetramer of FK506 binding protein 1B, which provides calcium to the myocardium. In addition, these regions are referred to as the N-terminal domain (domain I), central domain (domain II) and the channel region (domain III), depending on the potential physiological role for these *RYR2* mutation “hot spots” (1). The N-terminal domain comprises amino acids 77–466 and is the main binding site for FKBP12.6 subunit involved in channel activation (16–18). The central domain contains amino acids 2246–2534 and is conserved between species and isoforms (19, 20). Almost every region of the RYR2 protein is involved in channel modulation (21).

To date, over 150 different mutations have been identified that interfere with RYR2 ion channel function, and these mutations account for 70%–80% of CPVT cases (20, 22). However, Lehnart SE et al. reported that a heterozygous *p*.R2474S mutation of *RYR2* gene could cause seizures in mice, independent of cardiac arrhythmias, which is the first time to reveal the association of RYR2 with epilepsy, independent of CPVT (16). In a study, c.229 G > A/*p*.(Ala77Thr) in *RYR2* gene has been reported in a female patient with generalised epilepsy, which affects the N-terminal domain of the RYR2 receptor (8). Peng et al. identified a compound heterozygous missense mutation of *RYR2* (c.3248A > G and c.6779C > T) in a child with West syndrome (WS) (23). Recently, in a cohort of 292 cases with Benign epilepsy of childhood with centrotemporal spikes (BECTS), *RYR2* mutations were identified in 5 cases (24). In the present study, we reported a child with focal epilepsy carrying a mutation [c.12670G > T/*p*.(Glu4224*)] of *RYR2*. According to the EEG of the proband during the intermittent and ictal phases, he had focal seizures, instead of attacks due to heart abnormalities. In addition, the cases with *RYR2* mutation in the Ma et al. study were also confirmed as BECTS. Therefore, these cases demonstrated that *RYR2* may be a causative gene of focal epilepsy. It has been reported that the overall penetrance of *RYR2* mutation is 78% (39/50), eleven *RYR2* mutation carriers were considered to be phenotypically unaffected (7). It has been reported that there is a certain gender bias in the penetrance of *RYR2* mutation, male gender is a risk factor for syncope in patients with *RYR2* genotype, and most silent *RYR2* gene carriers in affected families are female (7, 25), which is in line with our results that the proband's mother as a RYR2 mutation carrier was phenotypically unaffected.

The mechanism by which *RYR2* mutations cause CPVT has been well investigated. It has been previously demonstrated that the pathological outcome of CPVT1 is either phosphorylation-induced leakage of RYR2 channels or Ca^2+^ overload of the sarcoplasmic reticulum (SR) (26–28). Abnormal release of Ca2^+^ could activate Na/Ca exchanger to depolarize the myocytes, leading to early or delayed afterdepolarization and arrhythmias (29, 30). Zhang X et al. established *RYR2*-CPVT-related mutation in hiPSC-CMs by CRISPR/Cas9 gene editing, and found that large sarcoplasmic reticulum Ca^2+^ leakage and smaller sarcoplasmic reticulum Ca^2+^ content were detected in cells carrying *p*.(Gln4201Arg) mutation (31). However, the pathogenic mechanism of *RYR2* mutations induced seizures has not been clarified. It is well known that calcium homeostasis is crucial to the stability of neuronal. In the present study, pathway enrichment analysis revealed that the genes correlated with *RYR2* were mainly involved in the calcium signaling pathway, revealing that RYR2 plays a critical role in calcium homeostasis regulation. However, no study has yet confirmed whether changes in the expression level of RYR2 in the brain directly lead to epilepsy. Herein, the novel mutation [c.12670G > T/*p*.(Glu4224*)] on *RYR2* may lead to truncation of the coding amino acid sequence. We hypothesized that the alteration of the physiologically important conformation of RYR2 protein may lead to RYR2 channel dysfunction, affecting the calcium signaling pathway and therefore causes seizures. This new mutant may shed light on the understanding of RYR2 function. We hope to be confirmed in more clinical samples, and further reveal its role and mechanism in epileptic seizures through structure-function analysis.

Sinus bradycardia in the resting state has been reported in some *RYR2* mutation carriers and in phenotypically affected CPVT patients (7). Recently, interictal arrhythmias was found in case 3 of BECTS identified with *RYR2* c.8574G > A, and case 5 with compound heterozygous missense mutation (*RYR2* c.7469T > C and c.12770G > A) presented sinus arrhythmia with mild cardiac structural abnormalities, and her father with no clinical symptoms, who carried a mutation (*RYR2* c.12770G > A), also exhibited sinus arrhythmia (24). However, the ECG of the other three probands showed no abnormalities, indicating the phenotypic diversity of *RYR2* mutation (24). Jiang T et al. reported a child with generalized epilepsy carried a heterozygous missense mutation of *RYR2* [c.14767A > T/*p*.(Met4923Leu)], showing abnormal sinus arrhythmia, ventricular extrasystoles, and paroxysmal ventricular tachycardia (32). In the present study, ECG showed sinus tachycardia in proband and his mother without phenotypic effects, rather than sinus bradycardia as reported in *RYR2* mutation carriers, but it was consistent with the case reported by Jiang. In addition, the color Doppler ultrasound confirmed that the proband and his mother had no cardiac structural abnormalities. Collectively, these cases suggest the heterogeneity of clinical phenotype caused by *RYR2* variants. Importantly, careful follow-up and specialized health consultation should be required for patients with *RYR2* mutation, who may present epilepsy and heart disease.

This study has some limitations. First, it remains unclear whether the delay in language development of the boy was associated with the identified *RYR2* mutation in the present study. Peng et al. Identified a compound heterozygous variant in RYR2 [c.3248A > G/*p*.(Glu1083Gly) and c.6779C > T/*p*.(Arg2260Leu)] in a case with WS exhibiting developmental delay or mental retardation (23). However, patients with *RYR2* mutations did not exhibit any psychomotor regression or impairment in the other studies. In our opinion, it may be due to the fact that the patient was younger at the time of consultation in the present study, and his language may be somewhat delayed, which may slowly return to normal with age. Additionally, the direct functional effects of the mutation [*RYR2* c.12670G > T/*p*.(Glu4224*)] was not examined to demonstrate the harmfulness. Therefore, larger numbers of cases and further studies are needed to confirm whether different *RYR2* mutations are related to more severe forms of epilepsy, and expand the whole spectrum of phenotype of RYR2 mutations, revealing the correlation between the variant and the patient's phenotype.

## Conclusions

In conclusion, we identified a novel *RYR2* mutation in a case of childhood-onset focal epilepsy, suggesting that *RYR2* mutations may cause epilepsy in humans. We propose that *RYR2* mutations may manifest with either CPVT or focal epilepsy, which may depend on the selective participation of *RYR2* receptors in the heart or brain.

## Data Availability

The authors acknowledge that the data presented in this study must be deposited and made publicly available in an acceptable repository, prior to publication. Frontiers cannot accept a manuscript that does not adhere to our open data policies.
