# Supplementary figures and images for: A novel mutation in ryanodine receptor 2 (RYR2) genes at c.12670G>T associated with focal epilepsy in a 3-year-old child
Source: Front Pediatr. 2022 Oct 19;10:1022268. doi: 10.3389/fped.2022.1022268 (PMC9627620; doi:10.3389/fped.2022.1022268)

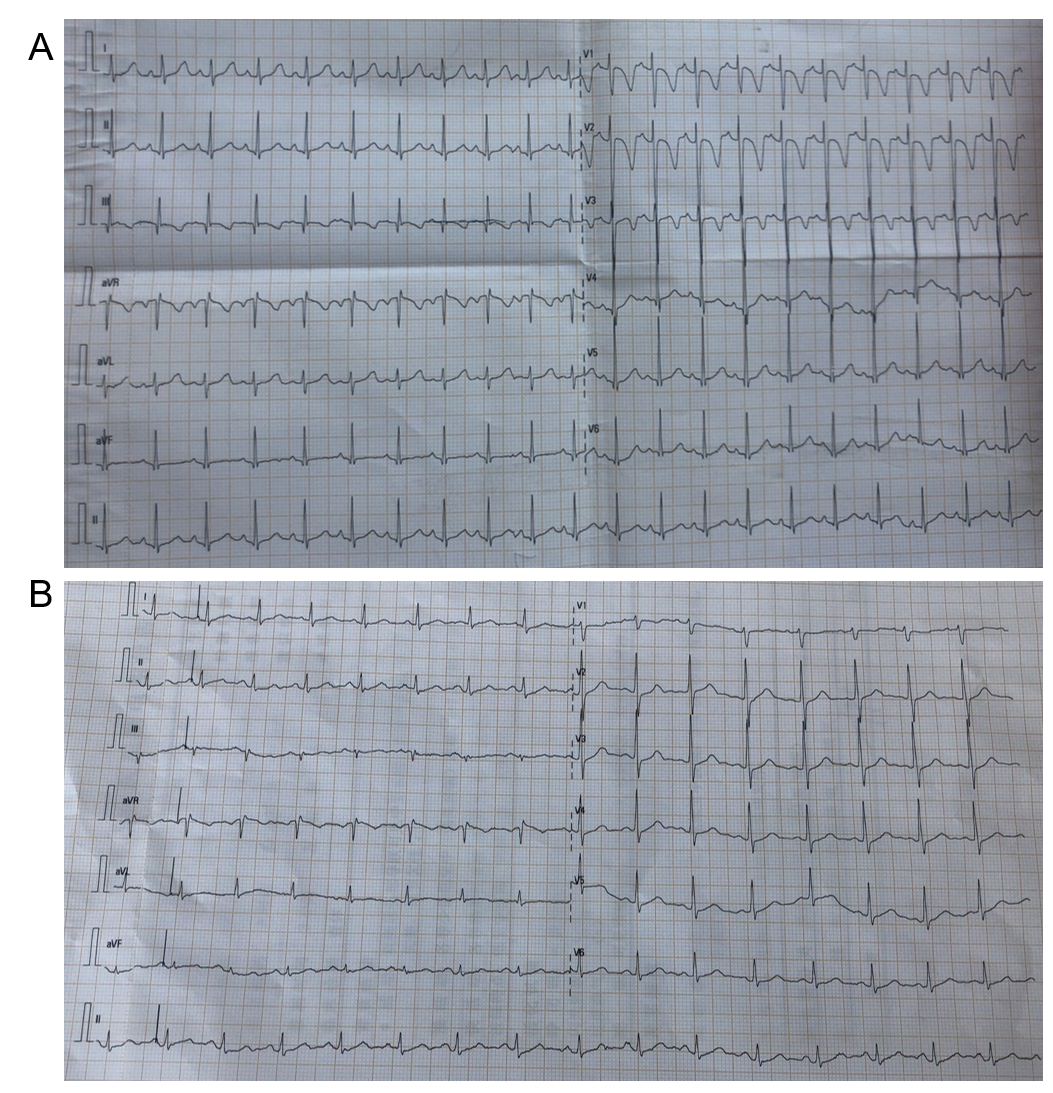

Supplement: Supplementary file 2 [file Image1.tif]
